# Identification of a novel nonsense *ATP2A2* gene variant in a patient with Darier’s disease flare following COVID-19 infection: A case report

**DOI:** 10.1097/MD.0000000000037335

**Published:** 2024-03-01

**Authors:** Linli Liu, Xiaotao Zheng, Qinglian Lu, Chunshui Yu

**Affiliations:** a Department of Dermatology, Suining Central Hospital, Suining, Sichuan, People’s Republic of China.

**Keywords:** *ATP2A2* gene, COVID-19, Darier disease, variant

## Abstract

**Rationale:**

Darier disease (DD) is a rare autosomal dominant disorder that primarily manifests as hyperkeratotic papules and itching. The underlying etiology of DD is pathogenic variation in the *ATP2A2* gene. However, this disease has a high penetrance but variable expressivity, indicating that patients inheriting the genotype may have different manifestations due to exogenous factors. Meanwhile, a few reports have documented that COVID-19 may be implicated in the flare of DD.

**Patient concerns:**

A 51-year-old man presented with keratotic papules and scaly erythematous rash on his trunk with pruritus after being infected with COVID-19. Laboratory test results were normal. Histological analysis revealed epidermal hyperkeratosis and intraepidermal lacunae containing dyskeratinized cells. Genetic analysis revealed a novel variant of *ATP2A2* (c.815G>A, p.Trp272*), which was considered pathogenic in this case.

**Diagnoses:**

The patient was diagnosed as having DD.

**Interventions:**

Oral acitretin and topical corticosteroid hormone ointments were used.

**Outcomes:**

The patient achieved complete resolution of symptoms during the 3-month follow-up period.

**Lessons:**

We revealed the first novel *ATP2A2* variant (c.815G>A, p.Trp272*) in the flare of DD following COVID-19 infection. Additionally, this pathogenic variant enriches the *ATP2A2* gene mutation spectrum.

## 1. Introduction

Darier disease (DD; OMIM #124200) is a rare autosomal dominant genodermatosis. The prevalence of DD is estimated to be approximately 1 in 30,000 to 100,000 individuals worldwide, affecting all ethnic groups and sexes equally.^[1]^ Clinically, DD commonly presents as keratotic papules accompanied by itching and malodor, primarily affecting seborrheic areas of the body.^[2]^ Histopathological analysis revealed acantholysis and dyskeratosis, hallmarks of this disorder.^[3]^ Previous studies have identified pathogenic mutations in *ATP2A2* as the underlying cause of DD.^[4]^ However, there are still gaps in our understanding of this condition, particularly regarding specific genetic variants and their association with disease severity and progression.

Recent studies have shed light on the dermatological manifestations of COVID-19. These skin manifestations in COVID-19 patients range from mild to severe and include urticarial rash, morbilliform rash, papulovesicular exanthem, and livedo reticularis.^[5]^ Notably, emerging reports have suggested a potential association between COVID-19 and flare-ups of preexisting DD.^[6]^ Therefore, it is crucial to understand the interplay between COVID-19 and DD. Exploring the relationship between these 2 conditions may provide valuable insights into the pathophysiology of DD and the effect of viral infections on disease.

Given the ongoing COVID-19 pandemic, it is crucial to recognize the potential impact of viral infections on dermatological disorders and their clinical outcomes. This study presents the case of a patient with DD flare following COVID-19 infection. The focus of this study was to detect novel genetic variants that contribute to the pathogenesis of the patient and investigate their association with disease flare following COVID-19 infection.

## 2. Case report

A 51-year-old Chinese male presented with a 4-month history of keratotic papules and a scaly erythematous rash on his trunk accompanied by pruritus. Prior to the onset of these symptoms, the patient had experienced a COVID-19 infection characterized by flu-like symptoms, including fever. Within a few days of the fever subsiding, a rash appeared on the trunk. The patient had a history of eczema, but had never encountered such severe symptoms in the past. He denied experiencing chills, cough, sore throat, diarrhea, or joint pain, and no similar rashes were reported by any household member.

**Figure 1.**
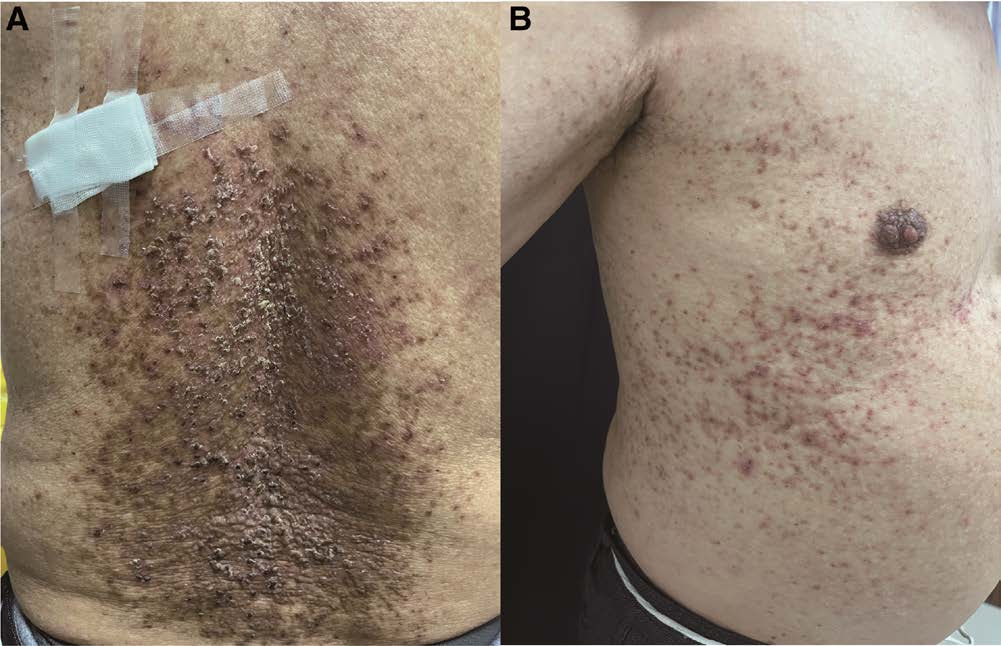
Photo of the patient demonstrating extensive keratotic papules and scaly erythematous rash on the patient’s trunk regions.

**Figure 2.**
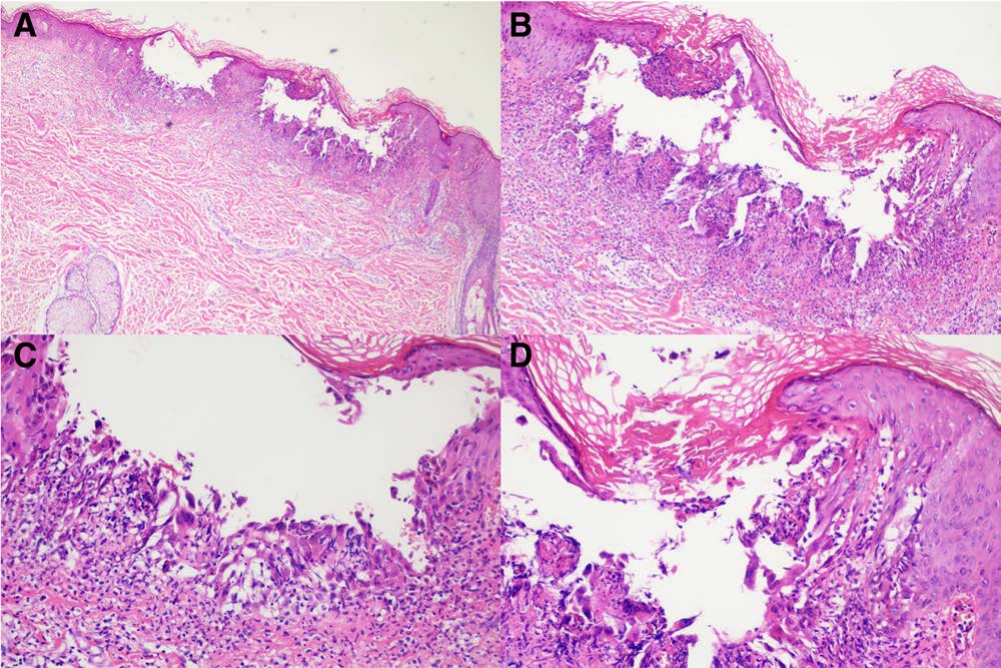
Biopsy showed epidermal hyperkeratosis and intraepidermal lacunae containing dyskeratinized cells. A, hematoxylin and eosin: ×40; B, hematoxylin and eosin: ×100; C and D, hematoxylin and eosin: ×200.

Physical examination revealed extensive keratotic papules and a scaly erythematous rash on the patient’s trunk (Fig. 1). The laboratory examination results were unremarkable. Histological analysis revealed epidermal hyperkeratosis and intraepidermal lacunae containing dyskeratinized cells (Fig. 2).

**Figure 3.**
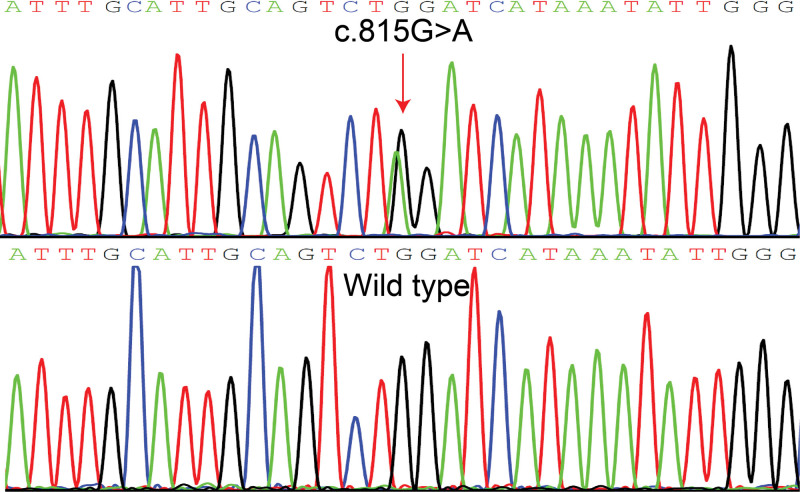
Sanger sequencing of the patient showed a heterozygous variant (c.815G>A, p.Trp272*) in the *ATP2A2* gene.

Next-generation sequencing identified a heterozygous variant (c.815G>A, p.Trp272*) in *ATP2A2*. This variant led to the substitution of tryptophan with a stop codon (p.Trp272*) (Fig. 3), resulting in functional changes in the protein. Importantly, this variant was absent from the patient’s family members and in a cohort of 100 unrelated controls. Its novelty was confirmed as it had not been documented in the ExAC, ESP, 1000G, or HGMD databases. According to the guidelines of the American College of Medical Genetics and Genomics (PSV1 + PM2 + PP2), this novel variant was classified as pathogenic.^[7]^ Based on these findings, the patient was diagnosed with DD and was subsequently treated with oral acitretin and topical corticosteroid hormone ointment for an appropriate duration. The patient achieved complete resolution of symptoms during the 3-month follow-up period.

This study was approved by the Ethics Committee of Suining Central Hospital and adhered to the tenets of the Declaration of Helsinki.

## 3. Discussion

This study describes a clinically diagnosed patient with DD harboring a novel heterozygous variant of *ATP2A2*. Clinically, DD manifests as hyperkeratotic papules primarily affecting the seborrheic area, which often should be distinguished from seborrheic dermatitis, acanthosis nigricans, Grover disease, and Hailey-Hailey disease.^[8]^ In this patient, dyskeratosis and acantholysis, which characterize DD, were observed on histopathology. The clinical manifestations and histopathology of the patient were consistent with the diagnosis of DD.^[9]^

Variants in *ATP2A2* have been identified as the underlying cause of DD.^[10]^ *ATP2A2* encodes sarco/endoplasmic reticulum Ca(2+)-ATPase isoform 2 (SERCA2), which plays a crucial role in maintaining calcium homeostasis within cells.^[11]^ *ATP2A2* pathogenic variants result in the production of dysfunctional SERCA2 protein, resulting in decreased ER Ca(2+) concentration in Darier keratinocytes, thus leading to impaired calcium transport and subsequent disruption of cell adhesion and differentiation.^[11]^ The specific genetic *ATP2A2* variant (c.815G>A, p.Trp272*) detected in this patient represents a novel nonsense mutation that alters protein function by substituting tryptophan with a stop codon at amino acid position 272 (p.Trp272*). This variant was considered pathogenic based on American College of Medical Genetics and Genomics guidelines.^[7]^ Presumably, this novel nonsense variant results in impaired SERCA2 function leading to aberrant junctional protein processing and impaired keratinocyte cohesion. This impairment potentially causes acantholysis or loss of connection between keratinocytes, as seen on pathology, which predominantly causes clinical manifestations in this case. Previous studies have suggested that nonsense mutations in *ATP2A2* are more common in DD patients. Ruiz-Perez reported that the majority (23/40) of mutations in *ATP2A2* are likely to result in nonsense-mediated RNA decay.^[12]^ Additionally, over 50% of the mutations lead to a premature termination codon, leading the authors to propose that haploinsufficiency is a common molecular mechanism for DD.^[4]^ Our investigation further supports this hypothesis. However, no genotype-phenotype relationship has been recognized for *ATP2A2* in DD,^[13]^ indicating high penetrance but variable expressivity. A possible reason could be that most SERCA2 protein functions were affected independently of the type or location of *ATP2A2* variants,^[13]^ and the phenotypic variability could also be due to additional environmental factors.^[14]^

DD is primarily triggered by exogenous factors, such as sun exposure, friction, or infection.^[15]^ The potential association between COVID-19 and flare-ups in DD is an intriguing area of research, as viral infections are known to trigger exacerbations in various dermatological conditions including DD.^[16]^ Some reports have suggested that SARS-CoV-2 may act as an environmental trigger for DD.^[17]^ The immune dysregulation and systemic inflammation induced by SARS-CoV-2 infection, including the cytokine storm-like response with tumor necrosis factor-alpha and interleukin-6, may downregulate *ATP2A2* mRNA levels. These pro-inflammatory cytokines and cellular immune responses can further disrupt calcium homeostasis, exacerbating the underlying pathophysiological mechanisms of DD through acantholysis and apoptosis.^[18]^ However, further research is needed to fully understand the precise mechanisms by which COVID-19 affects the course of DD.

The identification of the novel nonsense *ATP2A2* gene variant in this patient with COVID-19 provides valuable insights into the pathogenesis of DD associated with viral infections. The interaction between this genetic variant and inflammation and immune dysregulation induced by COVID-19 may have contributed to the observed disease flare-up in this patient. Understanding these mechanisms could lead to targeted therapeutic interventions and personalized management strategies for DD patients.

## 4. Conclusion

In conclusion, our study identified a novel nonsense *ATP2A2* variant in a patient with DD who experienced a disease flare following COVID-19 infection. This finding expands the gene spectrum associated with DD and highlights the potential influence of COVID-19 on disease flares. The precise mechanisms underlying the interaction between DD and COVID-19 require further investigation to better understand the pathophysiology and develop targeted interventions for patients with this condition.

## Acknowledgments

We thank the patient and his family members for their participation in this study.

## Author contributions

**Formal analysis:** Linli Liu.

**Investigation:** Qinglian Lu.

**Resources:** Xiaotao Zheng.

**Supervision:** Chunshui Yu.

**Visualization:** Qinglian Lu.

**Writing – original draft:** Linli Liu

**Writing – review & editing:** Linli Liu, Xiaotao Zheng, Qinglian Lu, Chunshui Yu.

## References

[1] Tavadia S Mortimer E Munro CS. Genetic epidemiology of Darier’s disease: a population study in the west of Scotland. Br J Dermatol. 2002;146:107–9. 11841374 10.1046/j.1365-2133.2002.04559.x

[2] Takagi A Kamijo M Ikeda S. Darier disease. J Dermatol. 2016;43:275–9. 26945535 10.1111/1346-8138.13230

[3] Engin B Kutlubay Z Erkan E. Darier disease: a fold (intertriginous) dermatosis. Clin Dermatol. 2015;33:448–51. 26051059 10.1016/j.clindermatol.2015.04.009

[4] Sakuntabhai A Burge S Monk S. Spectrum of novel ATP2A2 mutations in patients with Darier’s disease. Hum Mol Genet. 1999;8:1611–9. 10441323 10.1093/hmg/8.9.1611

[5] Genovese G Moltrasio C Berti E. Skin manifestations associated with COVID-19: current knowledge and future perspectives. Dermatology. 2021;237:1–12. 10.1159/000512932 PMC7801998 33232965

[6] Fukaura R Takeichi T Ebata A. COVID-19 infection- and vaccination-related exacerbation of Darier’s disease in a single patient. J Dermatol. 2023;50:833–6. 36651040 10.1111/1346-8138.16725

[7] Richards S Aziz N Bale S. Standards and guidelines for the interpretation of sequence variants: a joint consensus recommendation of the American College of Medical Genetics and Genomics and the Association for Molecular Pathology. Genet Med. 2015;17:405–24. 25741868 10.1038/gim.2015.30 PMC4544753

[8] Chyl-Surdacka K Borzęcki A Latifa J. Keratosis follicularis (Darier disease) – clinical characteristics and treatment – a review and update. Postepy Dermatol Alergol. 2023;40:337–40. 37545821 10.5114/ada.2022.124344 PMC10399679

[9] Rogner DF Lammer J Zink A. Darier and Hailey-Hailey disease: update 2021. J Dtsch Dermatol Ges. 2021;19:1478–502. 10.1111/ddg.14619 34661345

[10] Bachar-Wikström E Wikström JD. Darier disease – a multi-organ condition? Acta Derm Venereol. 2021;101:adv00430. 33606037 10.2340/00015555-3770 PMC9364244

[11] Savignac M Edir A Simon M. Darier disease: a disease model of impaired calcium homeostasis in the skin. Biochim Biophys Acta. 2011;1813:1111–7. 21167218 10.1016/j.bbamcr.2010.12.006

[12] Ruiz-Perez VL Carter SA Healy E. ATP2A2 mutations in Darier’s disease: variant cutaneous phenotypes are associated with missense mutations, but neuropsychiatric features are independent of mutation class. Hum Mol Genet. 1999;8:1621–30. 10441324 10.1093/hmg/8.9.1621

[13] Nellen RG Steijlen PM van Steensel MA. Mendelian disorders of cornification caused by defects in intracellular calcium pumps: mutation update and database for variants in ATP2A2 and ATP2C1 associated with Darier disease and Hailey-Hailey disease. Hum Mutat. 2017;38:343–56. 28035777 10.1002/humu.23164

[14] Leong IUS Stuckey A Ahanian T. Novel mutations in Darier disease and association to self-reported disease severity. PLoS One. 2017;12:e0186356. 29028823 10.1371/journal.pone.0186356 PMC5640244

[15] Suryawanshi H Dhobley A Sharma A. Darier disease: a rare genodermatosis. J Oral Maxillofac Pathol. 2017;21:321. 10.4103/jomfp.JOMFP_170_16 PMC5596695 28932054

[16] Drenovska K Schmidt E Vassileva S. Covid-19 pandemic and the skin. Int J Dermatol. 2020;59:1312–9. 32954488 10.1111/ijd.15189 PMC7537512

[17] Orsini D D’Arino A Pigliacelli F. SARS-CoV-2: an environmental trigger of Darier’s disease? J Dermatolog Treat. 2023;34:2242541. 37528796 10.1080/09546634.2023.2242541

[18] Dykes R Ergen E Martin T. Exacerbation of Darier’s disease with COVID-19. JAAD Case Rep. 2022;27:64–6. 35818536 10.1016/j.jdcr.2022.06.037 PMC9259054

